# Microbial Spectrum, Intraoperative Findings, and Postoperative Outcomes in Native Knee Joint Infections: A Retrospective Analysis

**DOI:** 10.3390/clinpract14060215

**Published:** 2024-12-16

**Authors:** Jonas Roos, Britta Mangels, Max Jaenisch, Matthias Dominik Wimmer, Thomas Martin Randau, Christian Prangenberg, Kristian Welle, Martin Gathen

**Affiliations:** Department of Orthopedics and Trauma Surgery, University Hospital of Bonn, 53127 Bonn, Germany

**Keywords:** native knee joint infections, *Staphylococcus aureus*, diagnostic criteria, septic arthritis, treatment modalities

## Abstract

**Background:** Native knee joint infections, while uncommon, present a serious condition predominantly instigated by bacteria such as *Staphylococcus aureus*. Without timely intervention, they can result in joint destruction or sepsis, with risk factors encompassing preexisting medical conditions and iatrogenic procedures. The diagnostic process includes a comprehensive patient history, clinical evaluation, laboratory testing, imaging studies, and microbiological investigations. Treatment typically involves joint aspiration and arthroscopy. This study aims to examine and establish correlations between diagnostic criteria and treatment modalities, enhancing the speed and specificity of future therapeutic strategies. **Materials and methods:** The present study is a retrospective cohort study conducted at a 1200-bed university clinic between 2007 and 2017, with an in-depth examination of patient details, symptoms, treatments, and outcomes. A scoring system was developed to classify the severity of knee joint impairment, categorizing patients on the basis of hospital stay duration, surgeries, and postoperative factors such as recurring symptoms, pain, and range of motion. **Results:** This study of 116 patients with knee joint infections revealed that clinical symptoms such as pain, swelling, and effusion are common but not definitive for diagnosis. Laboratory analysis revealed no significant differences in CRP or leukocyte counts between cultures positive or negative for pathogens. Hospital stay and disease severity are influenced by factors such as age, sex, presence of polyarthritis, neutrophil count, and type of pathogen, with higher weight and cortisone treatment associated with poorer outcomes. **Conclusions:** This study highlights the diagnostic challenges in native knee joint infections, revealing the need for comprehensive approaches given the nonspecificity of clinical symptoms and laboratory findings. This underscores the importance of advancing research through standardized methodologies and prospective studies to increase the accuracy of diagnosis and the effectiveness of treatment in this field.

## 1. Introduction

Native knee joint infection, which is characterized by the incursion of pathogenic microorganisms, predominantly bacteria, into the knee joint, causing inflammation, is distinct from the periprosthetic infections observed in knee joint arthroplasty [1]. These infections, though rare, can lead to severe joint damage, functional impairment, and potentially sepsis if not promptly diagnosed and treated [2].

In Western Europe, the incidence of septic arthritis ranges from 4–10 cases per 100,000 people, with mortality rates ranging between 7% and 15%, influenced by comorbidities [3,4]. The knee is the most commonly affected joint, accounting for 45% to 55% of cases [5]. Hematogenous spread is the leading cause, with predisposing factors such as diabetes mellitus and hematological diseases [6,7,8]. Iatrogenic interventions are significant exogenous factors [9].

The pathogen spectrum varies on the basis of the infection’s origin, patient age, and geographic location. *Staphylococcus aureus* is the most prevalent pathogen in Europe, with Streptococcus and Pneumococcus also commonly detected [10]. Different age groups show varied prevalence rates of pathogens, with children and elderly individuals being more likely to have Gram-negative rods and young adults showing Mycobacteria [3]. In immunocompromised individuals, fungi such as Candida and Sporothrix may occur [11].

Overall, the type of pathogen detected depends on the infection’s origin, with highly virulent bacteria such as *Staphylococcus aureus* in endogenous infections and less virulent organisms such as coagulase-negative staphylococci in exogenous infections [4]. A thorough and reproducible diagnostic approach is vital, including detailed patient history and clinical symptom assessment, routine laboratory tests focused on leukocyte count and C-reactive protein (CRP) levels, and imaging to exclude bone involvement [1,11,12,13,14]. Joint aspiration is the gold standard for diagnosis, with the aspirate’s macroscopic evaluation providing crucial insights [15,16,17]. This is also part of the guideline for diagnosis of septic arthritis in native joints (SANJO) [18]. Staging of the infection follows the Gächter classification, with arthroscopy and radiological imaging revealing specific characteristics of synovial inflammation [9,19]. Treatment typically involves arthroscopic cleansing to reduce the bacterial load [9,11].

The aim of this study was to analyze diagnostic parameters, treatment methods, and postoperative courses to correlate the accuracy and value of individual diagnostic components with intraoperative findings and outcomes, ultimately enabling quicker and more specific future diagnostic and therapeutic approaches.

## 2. Materials and Methods

### 2.1. Study Design

The present study is a retrospective cohort study conducted at an investigated hospital, which is a 1200-bed university clinic and the only Level I trauma center in a metropolitan area with a population of almost 1 million people. The clinic has further expertise in arthroplasty and spine surgery, is a center for geriatric traumatology, and is a certified cooperation partner of the German Cancer Society. Before proceeding, an ethical application was submitted and approved by the local Ethics Commission (Ref. No. 134/18).

### 2.2. Patient Collective

A total of 116 cases were included in the study. These subjects were selected on the basis of the criterion that they underwent surgical intervention for native knee joint infections at the specified hospital between 2007 and 2017. The data were systematically gathered via the Orbis system (Agfa HealthCare, Mortsel, Belgium).

### 2.3. Inclusion and Exclusion Criteria

The inclusion criteria specifically encompassed all patients with a confirmed infection of a native (non-arthroplasty) knee joint who required at least one surgical intervention on the affected limb to manage the infection. All patients with a cell count above 50,000, visible purulence in the aspirate, or a positive culture from the aspirate, as well as those showing signs of sepsis without any other infection focus besides the knee joint, were included and underwent surgical intervention. Patients with a history of trauma or previous knee injections were also included in the analysis. Exclusion criteria were defined as follows: patients were excluded if they had any form of joint replacement (e.g., knee arthroplasty) or foreign materials, such as osteosynthesis hardware, in the affected joint. Additionally, patients with primary malignancies or metastases in the affected limb were excluded to prevent confounding factors that could impact treatment outcomes or complicate the interpretation of infection-related findings.

### 2.4. Data Collection

The study’s data collection encompassed comprehensive patient details, including age, sex, height, weight, admission and discharge dates, the corresponding hospital stay duration, and the location of the affected limb. The general symptoms recorded were fever, pain, swelling, effusion, redness, and the presence of monoarthritis or polyarthritis. The knee joint aspiration was performed either through the lateral recess or the lateral knee portal, following verbal and written informed consent, under sterile conditions. The choice of approach depended on the physician’s experience. The aspirate was evaluated for its appearance, which could range from a purulent knee effusion, characterized by cloudy, white-yellow fluid, to a more typical synovial fluid appearance, which is viscous and nearly transparent. Additional patient data included diagnoses, medication intake (NSAIDs, anticoagulants, anti-rheumatic drugs), and laboratory parameters (C-reactive protein (CRP), leukocyte count, creatinine, hemoglobin (Hb), platelets, procalcitonin (PCT), D-Dimers). Comorbidities such as myocardial infarction, systolic heart failure, peripheral arterial occlusive disease, cerebrovascular disease, dementia, chronic lung disease, collagenosis, gastroduodenal ulcer disease, mild liver disease, and diabetes mellitus were also determined from the patient file. Data on knee joint puncture results, pre- and postoperative antibiotic and cortisone intake, and surgical interventions (arthroscopy, open surgery, combinations, or amputation) were also gathered.

Postoperatively, patients were contacted for follow-up information on their recovery. This included data on recurrent infections, additional surgeries, infection in other joints, renewed effusion in the affected joint, current pain levels on the VAS scale, rheumatologic diagnostics, family history, range of motion in the affected knee, and cortisone intake exceeding four weeks post discharge.

Patients were categorized into three groups on the basis of hospital stay duration and the nature and number of surgeries, reflecting the severity of their hospital stay. A mild course was defined as a stay under 14 days with intravenous (IV) antibiotic treatment and arthroscopy. A moderate course involved a stay of less than 21 days, one to two arthroscopies, or an open operation. A severe course was indicated by multiple surgeries and a stay exceeding 21 days. The criteria for discharging patients from inpatient treatment were declining infection rates, good clinical wound infection, and regular use of antibiotic therapy.

A scoring system was developed to assess the severity of knee joint impairment, factoring in recurring symptoms (0–3 points), pain (0–3 points), and range of motion (0–3 points). The total points determine the severity of the knee joint infection, ranging from 0 points for a favorable outcome to 9 points for a poor outcome characterized by constant pain, recurring symptoms, and significant knee joint impairment. This system was developed specifically for this study to provide a structured assessment of the severity of knee joint infection based on recurrent symptoms, pain, and range of motion. No prior validation was performed.

### 2.5. Statistical Analysis

The characteristics of the data are described as means with standard deviations (SDs) or medians with interquartile ranges (IQRs) for continuous variables and as frequency distributions with percentages for categorical variables. The normality distribution of continuous variables was tested using the Shapiro–Wilk test. For comparisons between patients with positive and negative culture results, *t* tests were used for normally distributed continuous variables, and the Mann—Whitney U test was used for nonnormally distributed continuous variables. Categorical variables were compared via chi-square tests. *p* values were corrected for multiple testing via the Bonferroni method. The significance level was set at *p* < 0.005. For specific analyses, differences in CRP levels between groups were assessed by t tests, leukocyte counts in blood samples were compared via the Mann—Whitney U test, and ANOVA was employed to analyze creatinine, hemoglobin (Hb), and platelet levels. T tests were used to compare leukocyte counts and neutrophil percentages in joint aspirates. Chi-square tests were used to identify factors associated with complicated hospital stays and to analyze the severity of disease progression. Binomial tests were conducted to assess the associations between specific clinical parameters and hospital stay outcomes. Correlation analyses were performed via chi-square tests to evaluate the impact of weight, polyarthritis, cortisone treatment, and synovialitis on patient outcomes.

## 3. Results

### 3.1. Clinical Symptoms

**Figure 1:**
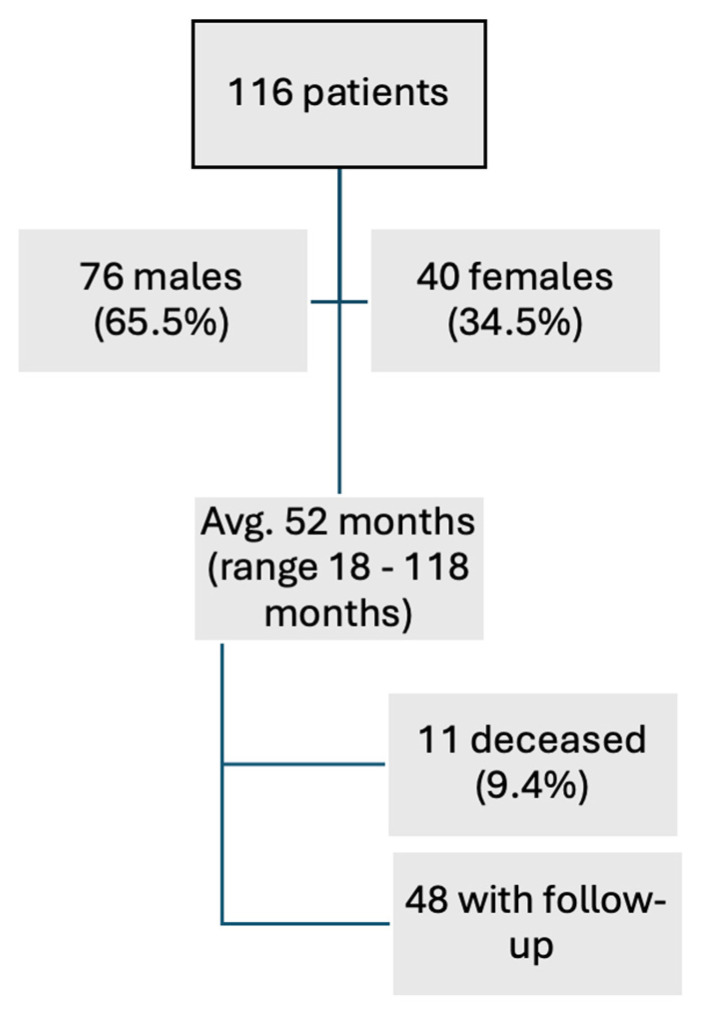
The figure shows a flowchart with the course of the study. Most patients were male (65.5%). The follow up was collected by contacting the patients via mail or phone. A total of 11 patients deceased.

**Figure 2:**
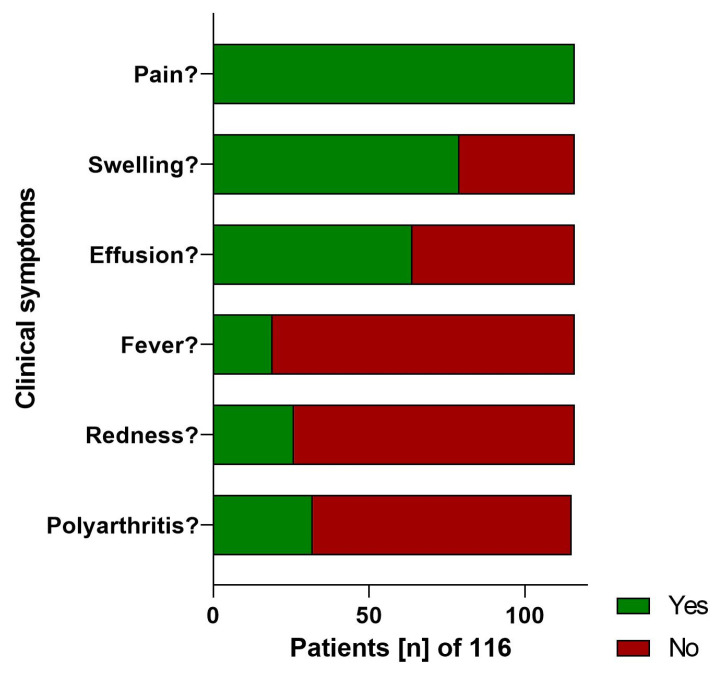
The figure shows the distribution and prevalence of clinical symptoms observed in patients with knee joint infections, categorized by frequency of occurrence.

The analysis incorporated data from 116 patients, comprising 76 (65.5%) males and 40 (34.5%) females. Of these, follow-up information was obtainable for 48 (42.3%) patients, with 11 (9.4%) reported deceased patients. Figure 1 provides an overview of the course of study. The remaining patients were either untraceable or declined to share further information. The average follow-up duration was 52 months, with a range of 18–118 months. The distribution of the occurrence of clinical symptoms is shown in Figure 2.

### 3.2. Laboratory Chemical Analysis

**Figure 3:**
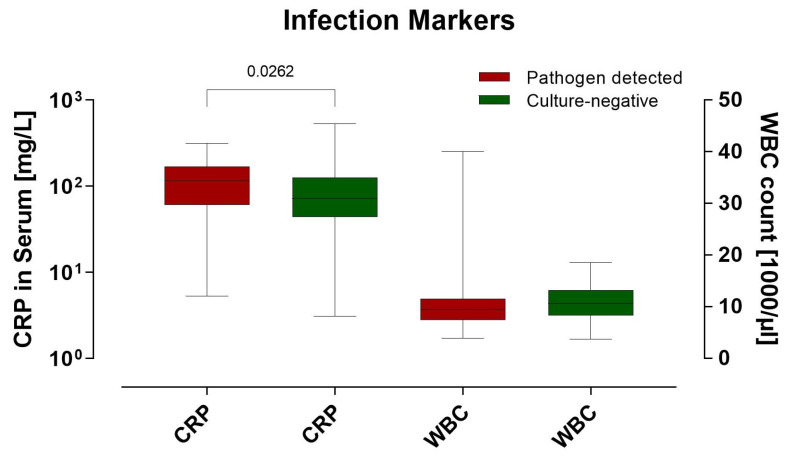
Illustration of the infection parameters depending on whether a pathogenic agent was detected or remained without evidence of microbial presence.

In patients with cultures positive for pathogenic agents, CRP levels were not significantly higher than those in patients with negative cultures (*p* = 0.026). Similarly, leukocyte counts in blood samples were not significantly different between groups with positive pathogen detection and those without. These findings are summarized in Figure 3.

Further analysis revealed no significant differences in creatinine, hemoglobin (Hb), or platelet levels between patients with and without pathogen detection via culture. The creatinine levels were within the normal range in both groups (0.8–1.2 mg/dL). The platelet counts were also the same in both groups and were in the normal range of approximately 200,000–380,000/µL. However, a notable observation was that Hb levels were slightly higher in the negative culture samples, ranging from 12 to 15 g/dL, than in the positive culture samples, which ranged from 11 to 13 g/dL.

### 3.3. Results of the Joint Aspirate

**Figure 4:**
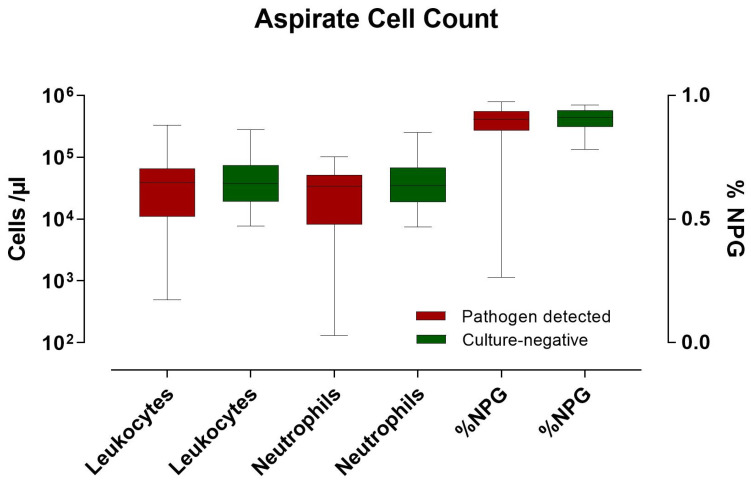
Cell count in the joint puncture aspirate. A comparison is made between leukocytes and neutrophils, as well as the percentage of neutrophils.

Elevated leukocyte counts of approximately 10,000 to nearly 100,000/µL were detected in the aspirate. The percentage of neutrophil granulocytes was also elevated at approximately 90%. No significant difference was detected between the two categories or between the detected pathogens and negative cultures (see Figure 4).

### 3.4. Parameters of the Complicated Hospital Stay

**Figure 5:**
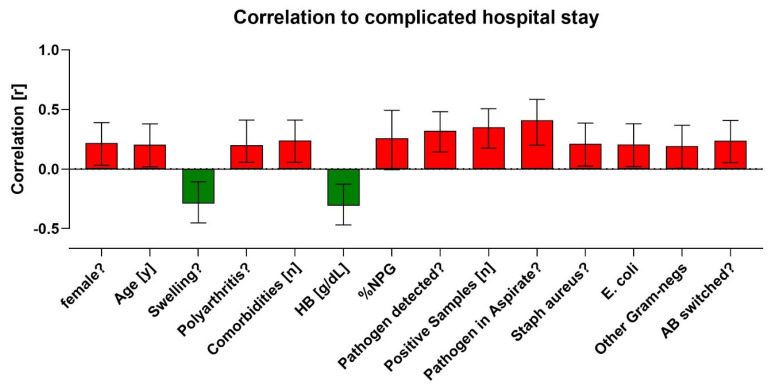
Compilation of parameters that promote or prevent a complicated hospital stay. The red-colored parameters correlate with a complicated hospital stay, whereas the green-colored parameters promote a favorable course of illness.

The parameters marked in red, including female sex, advanced age, the presence of polyarthrosis, various comorbidities, a high proportion of neutrophils, and the detection of pathogens (notably Staph. aureus, *E. coli*, or other Gram-negative pathogens) in cultures, other samples, and aspirates, as well as the necessity to change antibiotics, were identified as potential contributors to a complicated and extended hospital stay. Conversely, only two parameters, a swollen knee joint and an elevated hemoglobin (Hb) level, were associated with a more favorable hospital stay. Figure 5 provides a comprehensive summary of all the evaluated parameters.

### 3.5. Influence Parameters of Severity

Analysis revealed that among the 76 male patients with knee joint infections, approximately 45% experienced a mild course of the disease, whereas 82% of the 40 female patients exhibited moderate to severe progression. Additionally, a swollen knee was more commonly associated with a mild disease course, with less than 20% of mild cases presenting without knee swelling. In patients without swelling, moderate to severe disease was more prevalent. Monoarthritis generally indicates a milder course, whereas polyarthritis is associated with moderate disease progression.

The culture results also influenced the length of hospital stay. Negative cultures were typically associated with milder disease courses, a trend that was more pronounced if the joint puncture aspirate remained sterile. The detection of a pathogen in the aspirate led to a severe disease course in 40% of the patients. Specifically, the presence of *E. coli* was linked to severe disease in more than 60% of the cases and moderate disease in more than 35% of the cases. The absence of Staph. aureus in cultures corresponded to a mild course in 40% of the cases but also to severe progression in 20% of the cases. The detection of other Gram-negative pathogens predominantly resulted in severe disease.

**Figure 6:**
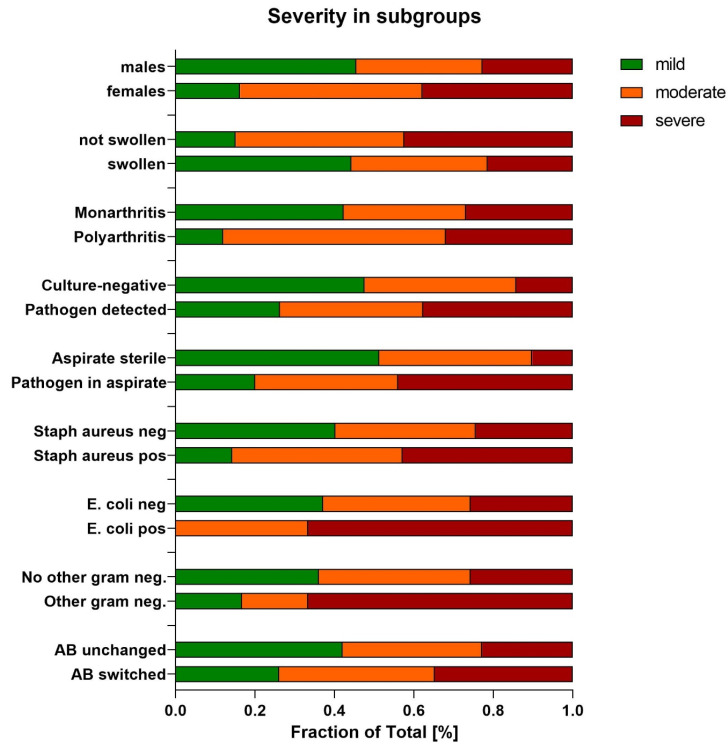
Degrees of severity of disease progression divided into subgroups (mild, moderate, and severe) in relation to the examined parameters.

With respect to antibiotic treatment, patients whose antibiotics were not changed typically experienced a mild disease course 40% of the time. Conversely, changing antibiotics was more often associated with moderate to severe disease progression. The classification of these various subgroups according to the parameters is depicted in Figure 6.

Patients with severe disease progression were observed to have significantly lower Hb values in laboratory tests compared with those with mild disease (*p* = 0.005), indicating that low Hb is a predictor of severe disease progression. Although not statistically significant, a high percentage of neutrophil granulocytes, over 90%, was noted in patients with severe progression. Age also played a role: younger patients (30–60 years) often had a mild course, whereas those aged 50–75 years frequently experienced severe progression. Patients aged 50–65 years commonly exhibited moderate hospital course severity, with no significant difference observed between the mild and severe progression groups (*p* = 0.0191).

The number of comorbidities was not significantly correlated with disease severity (*p* = 0.0324). However, patients with two to five comorbidities tended to have more severe disease courses than those with fewer or no comorbidities did, who mostly experienced mild to moderate disease courses. The absence of pathogens in cultures, or only a few positive cultures, generally corresponded with milder or moderate disease courses. In contrast, an increased number of positive cultures was significantly associated with severe progression (*p* < 0.005), indicating a greater likelihood of severe disease with one or more positive culture results.

## 4. Correlation

**Figure 7:**
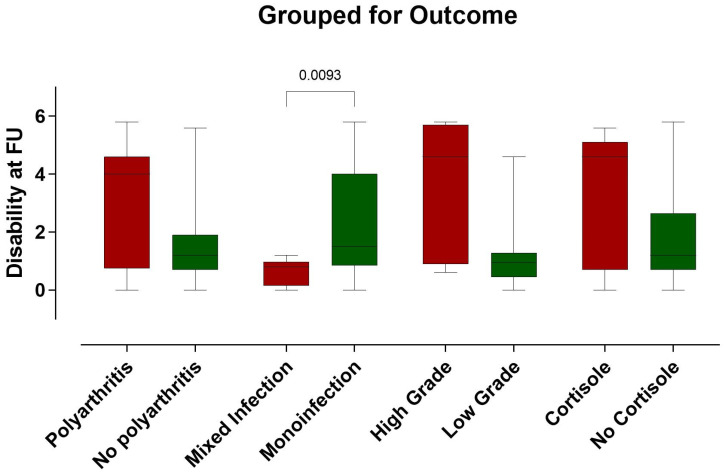
The figure shows the influence of polyarthritis, infections, high-grade synovitis and cortisone treatment on the outcome at follow-up (FU). The parameters for a poor result are shown graphically in red and those for a better result in green.

Analysis revealed that patients with greater weight, irrespective of BMI, and those with polyarthritis tended to have poorer outcomes. Additionally, treatment with cortisone, especially in the presence of multiple comorbidities, was generally associated with unfavorable outcomes. High-grade synovialitis, as classified by Krenn, was also correlated with poor prognosis. In contrast, patients with mixed infections typically experienced better outcomes than those with monoinfections did, indicating a significant difference in prognosis between the two groups. These findings suggest that polyarthritis leads to less favorable outcomes than monoarthritis does, and high-grade synovialitis is indicative of a worse prognosis than low-grade synovialitis is (see Figure 7).

**Figure 8:**
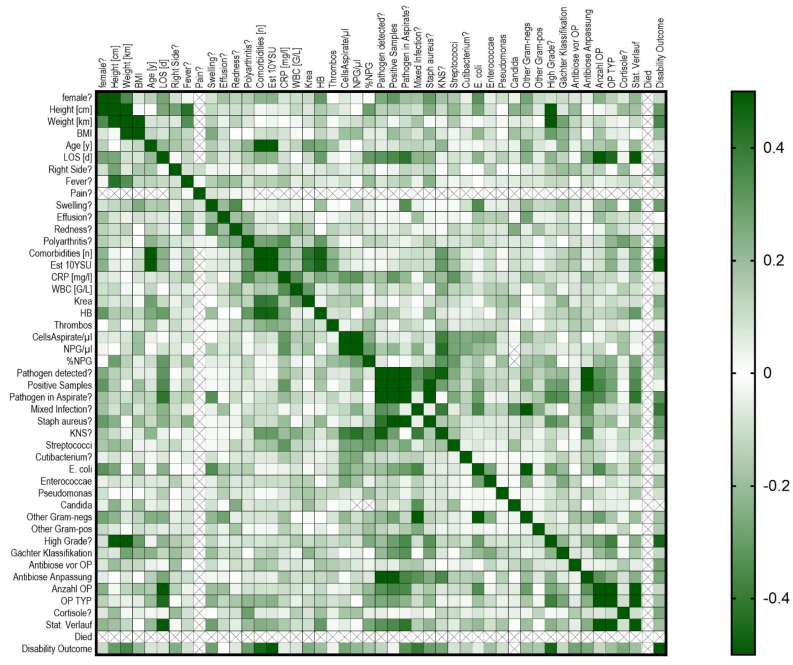
Correlations of risk factors and influencing factors for native knee joint infection.

In Figure 8, further correlations, including the relationship between patient age and the number of comorbidities, are explored. Older patients frequently had multiple comorbidities, whereas younger patients had fewer comorbidities. Additionally, samples positive for coagulase-negative staphylococci presented higher cell counts in the aspirate and a greater proportion of neutrophil leukocytes.

## 5. Discussion

The analysis of 116 patients with native knee joint infections revealed significant insights into disease progression and associated risk factors. Higher weight and the presence of polyarthritis were generally linked to poorer treatment outcomes. Cortisone treatment, particularly in patients with multiple comorbidities, was also correlated with unfavorable results. High-grade synovialitis, as classified by Krenn, was associated with poorer prognoses. In contrast, patients with mixed infections typically had better outcomes than those with monoinfections. Interestingly, patients with negative cultures or few positive cultures often experienced milder disease courses. Furthermore, older patients tended to have multiple comorbidities, whereas younger patients had fewer comorbidities. These findings underline the challenges in treatment and lay the groundwork for a detailed discussion of the implications and potential strategies for managing native knee joint infections.

The increasing incidence of native knee joint infections necessitates prompt and accurate diagnosis to prevent severe joint destruction and other complications, such as functional limitations or sepsis. This study reaffirms the importance of a multifaceted diagnostic approach, including patient history, physical examination, laboratory testing with blood culture collection, X-ray imaging, and optional sonography. This finding is consistent with previous research [2,11]. Our findings underscore the primary role of joint aspiration and cell count determination in diagnosis [15,20].

Our data revealed that pain in the affected knee joint was a universal symptom, aligning with prior studies that identified pain as a predominant symptom in patients with native knee joint infections [12]. However, the nonspecific nature of the clinical symptoms, such as swelling, effusion, redness, and fever, emphasizes the need for prompt knee joint puncture in suspected cases. Interestingly, our analysis revealed no significant difference in CRP levels or leukocyte counts between cultures with and without pathogen detection, challenging the reliability of these parameters in definitively diagnosing knee joint infections. Gerlach described that significantly elevated infection markers, such as leukocytes and CRP, are observed only in early infections [21]. These markers are neither specific nor sensitive and primarily serve as indicators or are used for monitoring disease progression and treatment response [11,22]. In cases of chronic joint infections, CRP levels are often unremarkable, which can lead to false-negative diagnoses. Moreover, in the presence of additional infection sites or rheumatoid arthritis, elevated CRP values are difficult to attribute to a specific cause [23].

The cell count in the aspirate, especially the presence of neutrophilic granulocytes, did not significantly differ between positive and negative cultures in our study. This observation aligns with Borzio et al., suggesting the need for larger sample sizes to identify significant differences [24]. The study highlights the lack of accuracy of most clinical and laboratory testing methods and found a synovial fluid WBC greater than 64.000 cells/µL as an optimal cutoff with 90% specificity but only 40% sensitivity.

Key findings from this study highlight the predictive nature of certain clinical markers. Knee joint swelling has emerged as an indicator of favorable outcomes, possibly because of early medical intervention. Elevated Hb levels also appeared to be positive predictors, echoing findings from hip joint infection studies and suggesting that low Hb might be a predictor of mortality [25]. This association aligns with prior findings that low Hb levels can indicate a more severe inflammatory response or systemic infection. For example, in the Laboratory Risk Indicator for Necrotizing Fasciitis (LRINEC) score, low Hb is considered one of the diagnostic indicators for severe infections like necrotizing fasciitis, underscoring the idea that decreased Hb levels may reflect underlying anemia of inflammation [26]. The favorable outcomes in mixed infections compared with monoinfections also point to the complexities in establishing effective antibiotic regimens [27]. The detection of Gram-negative pathogens, in particular the presence of *E. coli*, predominantly led to a severe course of the disease. According to current literature, Gram-negative pathogens are particularly common in intravenous drug use and in immunocompromised patients [28]. These factors also indicate a complicated course of the disease and require further investigation in the future, particularly with regard to the choice of empirical antibiotics. Nevertheless, *S. aureus* remains the most common in native knee joint infections [28,29].

Predisposing factors such as advanced age, comorbidities, and cortisone treatment were associated with more severe disease progression. The increased infection risk post cortisone injection highlights the necessity of considering patient history in diagnosis and treatment [30,31]. Particularly due to the limited evidence and the lack of long-term results [32], special caution should be exercised in patients with multiple comorbidities.

This study illustrates the challenges in diagnosing native knee joint infections, underscoring that no single diagnostic step is definitive. Chronic infections often present subtly, making early detection challenging. Our secondary hypothesis regarding the correlation of comorbidities with disease severity was confirmed, suggesting the need for heightened vigilance in patients with multiple risk factors [23], especially with the increasing number of post-arthroscopic infections and a high proportion of iatrogenic infections [33].

As is also common in the literature, the patients in our study underwent immediate surgical joint lavage [18,27]. There are also repeated studies showing that in the absence of clinical sepsis, immediate joint drainage does not appear to reduce the risk of secondary diseases compared to delayed drainage [34]. Further research is needed to further improve care and, in particular, to ensure the best possible treatment for at-risk patients.

Future research should focus on the role of CRP and explore other biomarkers for better diagnostic accuracy. This study highlights the imperative of timely diagnosis and treatment initiation for improved patient outcomes. In conclusion, this study identifies several parameters that could aid in earlier suspicion and assessment of knee joint infections, potentially guiding clinical decisions and improving prognoses.

This study is subject to several limitations. Its monocentric nature may restrict the generalizability of the findings. Additionally, the retrospective design of the research introduces constraints, particularly in terms of data collection. A prospective approach would have allowed for more focused and systematic gathering of information, potentially improving the quality and applicability of the results.

Future research could benefit from the implementation of standardized diagnostic protocols and criteria to ensure the consistency and replicability of findings. In this study, variations in diagnostic practices and the initiation of therapeutic interventions may have contributed to inconsistent outcomes.

Despite these limitations, this study offers valuable insights into the diagnosis and management of knee joint infections. These findings underscore the need for future investigations to consider these constraints and strive to address them, thereby enriching our understanding and treatment approaches in this field.

## Data Availability

Data available on request due to privacy/ethical restrictions.

## References

[1] Gerlach U.-J., Grimme C., Schoop R., Borree M. (2014). Gelenkinfekt—Eine Entität für die spezielle septische Chirurgie. Trauma Berufskrankh..

[2] Daynes J., Roth M.F., Zekaj M., Hudson I., Pearson C., Vaidya R. (2016). Adult Native Septic Arthritis in an Inner City Hospital: Effects on Length of Stay. Orthopedics.

[3] Loock J., Haustedt N., Wollenhaupt J. (2014). Septische Arthritis des Erwachsenen. Z. Rheumatol..

[4] Jabsen J., Seitz S. (2022). Septische Arthritis nativer Gelenke. Arthritis Rheuma.

[5] Zimmerli W., Borens O. (2013). 9 Die infektiöse Arthritis. Infekt. Bewegungsapparates.

[6] Koch K., Kohn D., Anagnostakos K. (2017). Structural Failure of a Modern Knee Tumor Megaendoprosthesis. Case Rep. Orthop..

[7] Schmidt H.G.K., Gerlach U.-J., Schoop R. (2005). Empyembehandlung. Trauma Berufskrankh..

[8] Seitz M. (2006). Rheumatologie in Kürze Klinisches Basiswissen für Die Praxis.

[9] Stutz G. (2005). Diagnostik und Therapie von Gelenkinfekten. SFA Arthrosk. Aktuell.

[10] Staphylococcus-aureus-Infektionen—Infektionen. https://www.msdmanuals.com/de-de/heim/infektionen/bakterielle-infektionen-grampositive-bakterien/staphylococcus-aureus-infektionen.

[11] Sendi P., Kühl R., Aeberli D., Zumstein M.A. (2017). Die septische Arthritis bei Erwachsenen. Swiss Med. Forum.

[12] Margaretten M.E., Kohlwes J., Moore D., Bent S. (2007). Does This Adult Patient Have Septic Arthritis?. JAMA.

[13] Hückstädt M., Hofmann G. (2017). Der Gelenkinfekt ohne Implantat. OP-Journal.

[14] Enderle E., Frosch K.-H. (2013). Stadiengerechte Therapie des Kniegelenkinfekts nach Arthroskopie. Oper. Orthop. Traumatol..

[15] AWMF-Leitlinie; Registernummer 029–006 (Intraartikuläre Punktionen und Injektionen: Hygienemaßnahmen). (n.d.). https://www.awmf.org/leitlinien/detail/ll/029-006.html.

[16] Ewerbeck V., Wentzensen A., Holz F., Krämer K.-L., Pfeil J., Sabo D. (2007). Standardverfahren in der Operativen Orthopädie und Unfallchirurgie.

[17] Der native Gelenkinfekt: “State of the Art”-Versorgung. https://www.universimed.com/ch/article/orthopaedie-traumatologie/der-gelenkinfekt-state-art-versorgung-48334.

[18] Ravn C., Neyt J., Benito N., Abreu M.A., Achermann Y., Bozhkova S., Coorevits L., Ferrari M.C., Gammelsrud K.W., Gerlach U.-J. (2023). Guideline for management of septic arthritis in native joints (SANJO). J. Bone Jt. Infect..

[19] Gächter A. (1988). Die Bedeutung der Arthroskopie beim Pyarthros. Hefte Unfallheilkd.

[20] Hettenkofer H.-J., Schneider M., Braun J. (2015). Rheumatologie: Diagnostik—Klinik—Therapie.

[21] Gerlach U.-J. (2017). Gelenkinfektion. OUP.

[22] Earwood J.S., Walker T.R., Sue G.J.C. (2021). Septic Arthritis: Diagnosis and Treatment. Am. Fam. Physician.

[23] Kemmerer M., Gramlich Y., Walter G., Hoffmann R. (2017). Infektionen der großen Gelenke–Diagnostik und therapeutische Strategie. Dtsch. Ärzteverlag OUP.

[24] Borzio R., Mulchandani N., Pivec R., Kapadia B.H., Leven D., Harwin S.F., Urban W.P. (2016). Predictors of Septic Arthritis in the Adult Population. Orthopedics.

[25] Kim B., Boukebous B., White D., Baker J.F. (2023). Septic arthritis of the native hip joint: A multi-pattern, multi-outcome disease. Eur. J. Orthop. Surg. Traumatol..

[26] Wong C.-H., Khin L.-W., Heng K.-S., Tan K.-C., Low C.-O. (2004). The LRINEC (Laboratory Risk Indicator for Necrotizing Fasciitis) score: A tool for distinguishing necrotizing fasciitis from other soft tissue infections*. Crit. Care Med..

[27] Ross J.J. (2017). Septic Arthritis of Native Joints. Infect. Dis. Clin. N. Am..

[28] Wu K.A., Kugelman D.N., Seidelman J.L., Seyler T.M. (2024). Native Joint Septic Arthritis. Antibiotics.

[29] Linke S., Thürmer A., Bienger K., Kleber C., Bellova P., Lützner J., Stiehler M. (2022). Microbiological pathogen analysis in native versus periprosthetic joint infections: A retrospective study. J. Orthop. Surg..

[30] Bonnaire F., Hohaus T., Lein T., Jaminet P. (2005). Bakterielle Gelenkinfektionen. OP-Journal.

[31] Tuqan A. (2014). Septic Arthritis after Intra-Articular Steroid Injection. Proc. UCLA Healthc..

[32] Ossendorff R., Thimm D., Wirtz D.C., Schildberg F.A. (2023). Methods of conservative intra-articular treatment for osteoarthritis of the hip and knee. Dtsch. Ärztebl. Int..

[33] Gunnlaugsdóttir S.L., Erlendsdóttir H., Helgason K.O., Geirsson Á.J., Thors V., Guðmundsson S., Gottfreðsson M. (2022). Native joint infections in Iceland 2003–2017: An increase in postarthroscopic infections. Ann. Rheum. Dis..

[34] Lauper N., Davat M., Gjika E., Müller C., Belaieff W., Pittet D., Lipsky B.A., Hannouche D., Uçkay I. (2018). Native septic arthritis is not an immediate surgical emergency. J. Infect..

